# The long-term effects of blood urea nitrogen levels on cardiovascular disease and all-cause mortality in diabetes: a prospective cohort study

**DOI:** 10.1186/s12872-024-03928-6

**Published:** 2024-05-16

**Authors:** Hongfang Liu, Xiaoqin Xin, Jinghui Gan, Jungao Huang

**Affiliations:** 1 Electrocardiography Department, Ganzhou Maternal and Child Health Hospital, Ganzhou, Jiangxi Province 341000 China; 2 Department of Clinical Laboratory, Ganzhou People’s Hospital, Ganzhou, Jiangxi Province 341000 China; 3 Department of Medical Genetic, Ganzhou Maternal and Child Health Hospital, Ganzhou, Jiangxi Province 341000 China

**Keywords:** Dietary intake, Blood urea nitrogen(BUN), Diabetes, Mortality, Nonlinear

## Abstract

**Background:**

The long-term effects of blood urea nitrogen(BUN) in patients with diabetes remain unknown. Current studies reporting the target BUN level in patients with diabetes are also limited. Hence, this prospective study aimed to explore the relationship of BUN with all-cause and cardiovascular mortalities in patients with diabetes.

**Methods:**

In total, 10,507 participants with diabetes from the National Health and Nutrition Examination Survey (1999–2018) were enrolled. The causes and numbers of deaths were determined based on the National Death Index mortality data from the date of NHANES interview until follow-up (December 31, 2019). Multivariate Cox proportional hazard regression models were used to calculate the hazard ratios (HRs) and 95% confidence interval (CIs) of mortality.

**Results:**

Of the adult participants with diabetes, 4963 (47.2%) were female. The median (interquartile range) BUN level of participants was 5 (3.93–6.43) mmol/L. After 86,601 person-years of follow-up, 2,441 deaths were documented. After adjusting for variables, the HRs of cardiovascular disease (CVD) and all-cause mortality in the highest BUN level group were 1.52 and 1.35, respectively, compared with those in the lowest BUN level group. With a one-unit increment in BUN levels, the HRs of all-cause and CVD mortality rates were 1.07 and 1.08, respectively. The results remained robust when several sensitivity and stratified analyses were performed. Moreover, BUN showed a nonlinear association with all-cause and CVD mortality. Their curves all showed that the inflection points were close to the BUN level of 5 mmol/L.

**Conclusion:**

BUN had a nonlinear association with all-cause and CVD mortality in patients with diabetes. The inflection point was at 5 mmol/L.

**Supplementary Information:**

The online version contains supplementary material available at 10.1186/s12872-024-03928-6.

## Background

Diabetes affects 1 in 11 individuals worldwide and is one of the major risk factors of death [1]. In 2021, 537 million adults lived with diabetes worldwide, and this number is expected to increase up to 700 million by 2045 [2]. Diabetes has several complications, and cardiovascular disease (CVD) is one of its main complications [3]. Therefore, the early identification of the modifiable risk factors to prevent mortality among patients with diabetes is of utmost importance.

Blood urea nitrogen (BUN) is a biochemical indicator derived from protein metabolism and excretion by the liver and kidneys [4] and is commonly used to measure protein intake and evaluate the status of renal function [5]. BUN levels are affected by several factors, including dehydration, protein intake, gastrointestinal bleeding, and liver disease; previous studies reported that BUN is not only used for evaluating the status of renal function [6]. Evidence from previous experiments suggested that the circulating urea levels have a direct impact on pancreatic beta cell function and lead to insulin secretory deficiency due to the production of oxidative stress and O-linked beta-N-acetylglucosamine in murine models [7, 8]. Based on the epidemiological evidence, a national prospective cohort study including 1,337,452 United States (US) veterans without diabetes conducted by Xie et al. concluded that BUN levels were positively associated with the occurrence of diabetes [9]. Similarly, the association was confirmed in a large Chinese cohort, and elevated BUN levels increased the prevalence of diabetes [10]. Moreover, Luo et al. found BUN levels on admission among patients with intracranial hemorrhage were associated with an increased risk of in-hospital death and 1-year mortality [11]. The results of a retrospective cohort of 2,008 Chinese participants shown higher BUN levels to serum albumin ratios were associated with an increased risk of short-term mortality [12]. However, existing prospective studies have not explored the role of BUN on long-term outcomes in patients with diabetes. This study aimed to explore the correlation of BUN levels with all-cause and CVD mortality in patients with diabetes using a nationally representative prospective cohort.

## Research design and methods

### Participants

Patients who participated in the National Health and Nutrition Examination Survey (NHANES) and those included in the 10 consecutive cycle datasets (1999–2000, 2001–2002, 2003–2004, 2005–2006, 2007–2008, 2009–2010, 2011–2012, 2013–2014, 2015–2016, and 2017–2018) were enrolled in the present study. The NHANES includes adults and children from 0 to 80 years of age. Briefly, the National Center for Health Statistics (NCHS) of the Centers for Disease Control and Prevention used complex, multistage, stratified methods to collect a representative sample of the US population. NHANES covers several variables, which are mainly related to the demographic, laboratory, dietary, examination, and important questionnaire data. These questionnaire variables were used by trained professionals during in-home and mobile examination center (MEC) interviews. More detailed information about NHANES is available online (https://www.cdc.gov/nchs/nhanes*).* The NHANES study was approved by the NCHS research ethics review board (protocols 98 − 12, 2005-06, 2011-17, and 2018-01). Patients aged ≥ 18 years who provided written informed consent upon enrollment were included in the study. Those who self-reported as pregnant were excluded (*n* = 1,305). Diabetes was ascertained according to the following criteria: [1] self-reported physician-diagnosed diabetes or current use of oral antihyperglycemic agents or insulin [2], a glycated hemoglobin A1c (HbA1c) level of ≥ 6.5% [3], a fasting blood glucose level of ≥ 7.0 mmol/L, and/or [4] a postprandial 2-h plasma glucose level of ≥ 11.1 mmol/L [13]. Patients with diabetes [1] with unavailable follow-up information (*n* = 19) [2], diagnosed with cancer at baseline (*n* = 1,739), and [3] with missing information on BUN levels (*n* = 717) were also excluded. Ultimately, 10,507 patients with diabetes were included in the analysis (Supplemental Fig. S1).

### Exposure and outcomes

BUN levels were measured using the enzymatic conductivity rate method, which is described in detail in the NHANES. The reference range for BUN levels was 3.1–9.5 mmol/L for adults. The outcomes were based on the mortality data of the National Death Index, which is an NCHS-centralized database, from the date of NHANES interview until follow-up (December 31, 2019) [14]. All underlying causes of death in the US population were precisely recorded following the International Statistical Classification of Diseases, 10th Revision (ICD-10). The ICD-10 codes I60–I69, I20–I51, I13, I11, and I00–I09 were used to determine the cause of CVD death, and malignant neoplasm was ascertained using the C00–C97 codes [15]. The follow-up interval was calculated using the difference between the last known date recorded in the mortality file for each individual and the date of the initial examination.

### Covariates

Sociodemographic information on age, marital status (widowed, divorced, married, separated, living with partner, and never married), race/ethnicity (other races, non-Hispanic White, other Hispanics, non-Hispanic Black, and Mexican–American), physical activity (inactive, active, and insufficiently active), smoking history (never, current, and former smokers), educational degree (< high school level, high school level, and > high school level), and alcohol intake (none, heavy, and low-to-moderate drinker) were obtained using standardized interview questionnaires. Household income was measured using the family income-to-poverty ratio (PIR). Patients who smoked ≥ 100 cigarettes, ≥ 100 cigarettes and then stopped smoking, and < 100 cigarettes were considered current, former, and never smokers, respectively [2]. During leisure time, patients who did not perform physical exercise were categorized as “inactive.” Those who engaged in moderate activity with metabolic equivalents of 3–6 at least five times each week or in strenuous exercise with a metabolic equivalent of > 6 at least thrice per week, which met the recommended levels of leisure–time activity, were categorized as “active”; those who were neither inactive nor did not meet the criteria for the active group were categorized as “insufficiently active” [16]. Alcohol status was determined based on the 24-h dietary intake measured during the MEC interview. Men consuming 0.1–27.9 g of alcohol per day and women consuming 0.1–13.9 g of alcohol per day were classified as moderate drinkers, men consuming > 28 g of alcohol per day and women consuming > 14 g of alcohol per day were classified as heavy drinkers, and patients who consumed 0 g of alcohol per day were classified as nondrinkers. Healthy Eating Index 2015 (HEI-2015) was introduced to measure the diet quality [17]. Body mass index (BMI) was subdivided into three categories (≥ 30.0, 25.0–29.9, and < 25.0 kg/m^2^) [2]. The criteria for diagnosing baseline hypertension were as follows: [1] taking antihypertensive medications or [2] having a systolic blood pressure of ≥ 130 mmHg and/or a diastolic blood pressure of ≥ 80 mmHg [18]. The diagnosis of dyslipidemia was based on the following conditions: [1] high-density lipoprotein levels of < 40 mg/dL in men and < 50 mg/dL in women [2], a total cholesterol (TC) level of ≥ 200 mg/dL [3], a low-density lipoprotein level of ≥ 130 mg/dL [4], a triglyceride level of ≥ 150 mg/dL, and/or [5] taking lipid-lowering medications [19]. Data on diabetes duration and use of diabetes medications, including oral hypoglycemic drugs and insulin, were collected when the patients were interviewed during medical visits. The homeostatic model assessment of insulin resistance (HOMA-IR) was calculated using the formula adopted in previous studies [20]. A 24-h recall interview was performed to collect information on the patient’s daily diet [21]. We calculated the total energy intake (TEI) using the US Department of Agriculture Automated Multiple-Pass Method [22]. The baseline CVD was diagnosed by a professional physician. The baseline levels of biochemical markers, including serum cotinine, serum calcium, serum TC, urine albumin, and serum triglycerides, were strictly analyzed according to the laboratory standards. Further experimental details were also documented in the NHANES. The duration of diabetes and the medications used were obtained through interviews during medical visits.

### Statistical analyses

Free Statistics software version 1.4 was used to perform statistical analysis, which was based on the R statistical software package (http://www.R-project.org, the R Foundation) [23]. Categorical and normally distributed continuous variables were presented as numbers (*n*) with percentages (%) and as means with standard deviations, and nonnormally distributed continuous variables were presented as medians with interquartile ranges. The patients were divided into quarters (Q1–Q4) according to BUN values. The difference between the four groups was compared by one-way ANOVA tests (continuous variables with normal distribution and homogeneity of variance), Kruskal-Wallis test (continuous variables with nonnormal distribution), and χ^2^ test (categorical variables). A *P*-value of < 0.05 was considered significant. Person-time was calculated based on either [1] the interval between the date of NHANES interview and death and [2] the interval between the date of NHANES interview and follow-up (December 31, 2019), whichever came first. Kaplan–Meier analysis and log-rank test were used in the univariate survival analysis. The hazard ratios (HRs) and 95% confidence intervals (CIs) of CVD, cancer, and all-cause mortality were calculated using the multivariate Cox proportional hazards regression models. In Model 1, none of the variables were adjusted. Variables such as gender, race/ethnicity, and age were included in Model 2. Model 3 was fully adjusted for gender, race/ethnicity, age, PIR; educational status; marital status; BMI; HbA1c and serum TC levels; diabetes medication use; diabetes duration; insulin therapy; serum cotinine, calcium, serum triglyceride, urine albumin, magnesium, and vitamin D levels; alcohol intake; multivitamin supplement use; kidney disease; TEI; HEI; HOMA-IR; hypertension; hyperlipidemia; CVD; smoking status; and physical activity.

Subgroup analysis was performed according to age (≤ 60 or > 60 years), BMI level (< 30 or ≥ 30 kg/m^2^), gender, race/ethnicity (others or non-Hispanic White), alcohol intake status, smoking status, diabetes duration (≤ 3 or > 3 years), physical activity, and HbA1c level (< 7% or ≥ 7%). To explore the potential interaction effects between subgroups, likelihood ratio tests were conducted to assess the corresponding multiplicative interaction terms, and P values were also calculated. Moreover, a series of sensitivity analyses were performed. First, patients who died within 2 years after the baseline examination were excluded to reduce the possibility of reverse causality. Second, considering that dietary protein intake plays a critical role in the BUN levels, the sensitivity analysis was additionally adjusted for protein intake. Third, severe liver disease may decrease the BUN levels; therefore, we excluded patients with liver disease. Fourth, as part of the sensitivity analysis, we also determined the association of BUN with mortality among patients with normal BUN levels (3.1–9.5 mmol/L). Fifth, considering that other potential confounding factors may still exist, the inverse probability of treatment weighting (IPTW) method was used to compare the Q1 and Q4 groups. Covariates used in the model were gender, age, BMI, race/ethnicity, PIR, alcohol intake, HOMA-IR, smoking status, HbA1c level, physical activity, diabetes duration, and kidney disease. We used restricted cubic splines(RCS) with three knots to visualize the dose-response association between BUN and mortality. In addition, generalized additive models were used to explore the potential nonlinear association of BUN levels with mortality. When a nonlinear association was found in the models, a recursive algorithm was used to calculate the inflection points in order to determine the link between BUN and mortality; a two-piecewise linear regression was also performed.

## Results

The baseline characteristics of the study population are listed in Table 1 according to the BUN level quartiles. Out of the 10,570 patients with diabetes from the NHANES 1999–2018, only 4,963 (47.2%) women were included. The average age of patients was 58.1 ± 15.4 years. The median (interquartile range) BUN level was 5 (3.93–6.43) mmol/L. A higher BUN level was frequently reported among patients who were male, older age, Caucasian, had middle family income, were married and inactive, and had higher HOMA-IR level, shorter diabetes duration, and hypertension and hyperlipidemia. During an average follow-up period of 8.24 years, 2,441 deaths were documented, 858 of which were due to cardiovascular disease and 398 due to cancer. Kaplan–Meier analysis indicated an association between lower BUN levels and lower all-cause, CVD, and cancer mortality rates among patients with diabetes (Supplemental Fig. S2). In the fully adjusted model, BUN was associated with all-cause and CVD mortality rates, and the association of BUN with cancer mortality disappeared (Table 2). Compared with the lowest quartile of BUN levels, the HRs of all-cause and CVD mortality in the highest quartile group were 1.35 (95% CI, 1.18–1.54) (P_trend_ < 0.001) and 1.52 (95% CI, 1.2–1.92) (P_trend_ < 0.001) in the fully adjusted model, respectively. With a one-unit increment in BUN levels, the HRs of all-cause and CVD mortality were 1.07 (1.06–1.08) and 1.08 (1.06–1.1), respectively. Moreover, the stratified analyses indicated that these associations remained unchanged (Supplemental Figs. S3 and S4). Meanwhile, there was no association found between BUN and all-cause and CVD mortalities among the physically active or alcohol intake groups, largely due to the limited sample size. No significant interaction effects were observed between the subgroups.

**Table 1:** Baseline characteristics of patients with type 2 diabetes, according to quartiles of BUN

Variables	Total	Q1 < 3.93	Q2 3.93-5.00	Q3 5.00-6.43	Q4 > 6.43	*P*
Participants, n	10,507	2391	2567	2738	2811	
Sex, n (%)						< 0.001
Female	4963 (47.2)	1385 (57.9)	1201 (46.8)	1155 (42.2)	1222 (43.5)	
Male	5544 (52.8)	1006 (42.1)	1366 (53.2)	1583 (57.8)	1589 (56.5)	
Age, Mean ± SD	58.1 ± 15.4	50.0 ± 15.0	54.3 ± 15.3	59.6 ± 13.9	66.9 ± 12.0	< 0.001
Race, n (%)						< 0.001
Mexican–American	2280 (21.7)	560 (23.4)	577 (22.5)	592 (21.6)	551 (19.6)	
Non-Hispanic White	3781 (36.0)	703 (29.4)	828 (32.3)	1037 (37.9)	1213 (43.2)	
Non-Hispanic Black	2357 (22.4)	670 (28)	604 (23.5)	529 (19.3)	554 (19.7)	
Other Hispanics	1035 (9.9)	226 (9.5)	262 (10.2)	291 (10.6)	256 (9.1)	
Other races	1054 (10.0)	232 (9.7)	296 (11.5)	289 (10.6)	237 (8.4)	
PIR, n (%)						< 0.001
≤ 1.30	3831 (36.5)	1030 (43.1)	931 (36.3)	908 (33.2)	962 (34.2)	
1.31–3.50	4053 (38.6)	862 (36.1)	949 (37)	1068 (39)	1174 (41.8)	
≥ 3.50	2623 (25.0)	499 (20.9)	687 (26.8)	762 (27.8)	675 (24)	
Education_level, n (%)						< 0.001
< high school level	3827 (36.4)	857 (35.8)	879 (34.2)	969 (35.4)	1122 (39.9)	
High school level	2410 (22.9)	573 (24)	580 (22.6)	618 (22.6)	639 (22.7)	
> high school level	4270 (40.6)	961 (40.2)	1108 (43.2)	1151 (42)	1050 (37.4)	
Marital_status, n (%)						< 0.001
Never married	1168 (11.1)	413 (17.3)	324 (12.6)	232 (8.5)	199 (7.1)	
Married	5783 (55.0)	1180 (49.4)	1436 (55.9)	1626 (59.4)	1541 (54.8)	
Lives with partner	1210 (11.5)	300 (12.5)	297 (11.6)	313 (11.4)	300 (10.7)	
Widowed	552 (5.3)	179 (7.5)	157 (6.1)	137 (5)	79 (2.8)	
Divorced	407 (3.9)	126 (5.3)	101 (3.9)	85 (3.1)	95 (3.4)	
Separated	1387 (13.2)	193 (8.1)	252 (9.8)	345 (12.6)	597 (21.2)	
BMI, kg/m2	31.6 ± 7.5	32.2 ± 8.0	31.7 ± 7.3	31.4 ± 7.4	31.3 ± 7.3	< 0.001
HbA1c, n (%)						< 0.001
< 7	7211 (68.6)	1671 (69.9)	1852 (72.1)	1878 (68.6)	1810 (64.4)	
≥ 7	3296 (31.4)	720 (30.1)	715 (27.9)	860 (31.4)	1001 (35.6)	
Serum total cholesterol, mg/dL	193.6 ± 46.4	195.5 ± 44.4	197.1 ± 46.8	195.1 ± 47.0	187.3 ± 46.5	< 0.001
Diabetes medication use, n (%)						< 0.001
No	4133 (39.3)	1030 (43.1)	1086 (42.3)	1027 (37.5)	990 (35.2)	
Yes	6374 (60.7)	1361 (56.9)	1481 (57.7)	1711 (62.5)	1821 (64.8)	
Diabetes duration, years						< 0.001
≤ 3 years	9397 (89.4)	2225 (93.1)	2396 (93.3)	2492 (91)	2284 (81.3)	
3–10 years	543 (5.2)	92 (3.8)	96 (3.7)	123 (4.5)	232 (8.3)	
≥ 10 years	567 (5.4)	74 (3.1)	75 (2.9)	123 (4.5)	295 (10.5)	
Insulin therapy, n (%)						< 0.001
No	9092 (86.5)	2183 (91.3)	2359 (91.9)	2409 (88)	2141 (76.2)	
Yes	1415 (13.5)	208 (8.7)	208 (8.1)	329 (12)	670 (23.8)	
Serum cotinine, Mean ± SD	51.7 ± 126.0	78.3 ± 148.5	52.0 ± 124.4	45.3 ± 118.9	35.1 ± 108.5	< 0.001
Calcium, Mean ± SD	2.4 ± 0.1	2.3 ± 0.1	2.4 ± 0.1	2.4 ± 0.1	2.4 ± 0.1	0.01
Serum triglycerides, mg/dL	180.9 ± 178.0	171.8 ± 158.3	185.0 ± 216.5	184.6 ± 181.5	181.2 ± 148.9	0.032
Urine albumin, Mean ± SD	128.4 ± 682.4	55.5 ± 303.4	56.2 ± 353.1	90.0 ± 560.8	293.6 ± 1097.6	< 0.001
Magnesium, Mean ± SD	275.7 ± 141.1	270.9 ± 149.9	283.7 ± 144.9	283.5 ± 137.6	264.9 ± 132.0	< 0.001
VitaminD, Mean ± SD	4.3 ± 5.3	3.7 ± 4.5	4.2 ± 4.9	4.4 ± 5.2	4.8 ± 6.3	< 0.001
Alcohol intake, n (%)						< 0.001
Nondrinker	8669 (82.5)	1926 (80.6)	2093 (81.5)	2252 (82.2)	2398 (85.3)	
Low-to-moderate drinker	691 (6.6)	150 (6.3)	177 (6.9)	181 (6.6)	183 (6.5)	
Heavy drinker	1147 (10.9)	315 (13.2)	297 (11.6)	305 (11.1)	230 (8.2)	
Multivitamin supplements use, n (%)						0.255
No	10,393 (98.9)	2374 (99.3)	2537 (98.8)	2705 (98.8)	2777 (98.8)	
Yes	114 (1.1)	17 (0.7)	30 (1.2)	33 (1.2)	34 (1.2)	
Kidney disease, n (%)						< 0.001
No	9893 (94.2)	2329 (97.4)	2508 (97.7)	2637 (96.3)	2419 (86.1)	
Yes	614 (5.8)	62 (2.6)	59 (2.3)	101 (3.7)	392 (13.9)	
TEI, kcal, Mean ± SD	1934.1 ± 928.4	1972.7 ± 989.5	2019.9 ± 962.5	1977.5 ± 916.5	1780.4 ± 831.9	< 0.001
HEI-2015, Mean ± SD	51.3 ± 13.6	49.9 ± 13.5	50.6 ± 13.8	51.6 ± 13.5	52.8 ± 13.4	< 0.001
HOMA-IR, Mean ± SD	7.7 ± 12.1	7.2 ± 10.1	7.1 ± 10.1	7.4 ± 11.5	8.8 ± 15.3	< 0.001
Hypertension, n (%)						< 0.001
No	3761 (35.8)	1105 (46.2)	1100 (42.9)	934 (34.1)	622 (22.1)	
Yes	6746 (64.2)	1286 (53.8)	1467 (57.1)	1804 (65.9)	2189 (77.9)	
Hyperlipidemia, n (%)						< 0.001
No	1582 (15.1)	409 (17.1)	419 (16.3)	403 (14.7)	351 (12.5)	
Yes	8925 (84.9)	1982 (82.9)	2148 (83.7)	2335 (85.3)	2460 (87.5)	
Baseline CVD, n (%)						< 0.001
No	8391 (79.9)	2087 (87.3)	2230 (86.9)	2203 (80.5)	1871 (66.6)	
Yes	2116 (20.1)	304 (12.7)	337 (13.1)	535 (19.5)	940 (33.4)	
Smoke status, n (%)						< 0.001
Never smokers	5376 (51.2)	1180 (49.4)	1369 (53.3)	1398 (51.1)	1429 (50.8)	
Former smokers	3235 (30.8)	528 (22.1)	715 (27.9)	918 (33.5)	1074 (38.2)	
Current smokers	1896 (18.0)	683 (28.6)	483 (18.8)	422 (15.4)	308 (11)	
Physical activity, n (%)						< 0.001
Inactive	6876 (65.4)	1503 (62.9)	1605 (62.5)	1787 (65.3)	1981 (70.5)	
Insufficiently active	2564 (24.4)	606 (25.3)	641 (25)	671 (24.5)	646 (23)	
Active	1067 (10.2)	282 (11.8)	321 (12.5)	280 (10.2)	184 (6.5)	

PIR, poverty income ratio; BMI, Body mass index; TEI, total energy intake; HEI-2015, Healthy Eating Index 2015; HOMA-IR, homeostatic model assessment of insulin resistance; CVD, cardiovascular disease

**Table 2:** Associations of BUN level with all-cause, CVD, and cancer mortality in patients with diabetes from the NHANES 1999–2018 cohort

Characteristic	Q1 < 3.93	Q2 3.93-5.00	Q3 5.00-6.43	Q4 > 6.43	Per 1 mmol/l increment	*P* for trend
All-cause mortality						
Incidence/person-years	339/21,896	399/22,035	598/22,801	1105/19,869	2441/86,601	
Model 1	1 (ref)	1.19 (1.03 ∼ 1.38)	1.7 (1.49 ∼ 1.94)	3.78 (3.35 ∼ 4.27)	1.15 (1.14 ∼ 1.16)	< 0.001
Model 2	1 (ref)	0.86 (0.74 ∼ 1)	0.91 (0.8 ∼ 1.05)	1.35 (1.19 ∼ 1.54)	1.1 (1.09 ∼ 1.11)	< 0.001
Model 3	1 (ref)	0.99 (0.85 ∼ 1.15)	1.02 (0.89 ∼ 1.18)	1.35 (1.18 ∼ 1.54)	1.07 (1.06 ∼ 1.08)	< 0.001
CVD mortality						
Incidence/person-years	102/21,896	116/22,035	218/22,801	422/19,869	858/86,601	
Model 1	1 (ref)	1.15 (0.88 ∼ 1.5)	2.06 (1.63 ∼ 2.61)	4.78 (3.85 ∼ 5.94)	1.16 (1.15 ∼ 1.17)	< 0.001
Model 2	1 (ref)	0.82 (0.62 ∼ 1.07)	1.08 (0.85 ∼ 1.37)	1.62 (1.29 ∼ 2.04)	1.11 (1.09 ∼ 1.13)	< 0.001
Model 3	1 (ref)	0.93 (0.71 ∼ 1.22)	1.19 (0.94 ∼ 1.52)	1.52 (1.2 ∼ 1.92)	1.08 (1.06 ∼ 1.1)	< 0.001
Cancer						
Incidence/person-years	74/21,896	82/22,035	114/22,801	128/19,869	398/86,601	
Model 1	1 (ref)	1.12 (0.82 ∼ 1.53)	1.49 (1.11 ∼ 2)	2.01 (1.51 ∼ 2.67)	1.09 (1.06 ∼ 1.12)	< 0.001
Model 2	1 (ref)	0.8 (0.58 ∼ 1.1)	0.79 (0.58 ∼ 1.06)	0.71 (0.53 ∼ 0.96)	0.99 (0.94 ∼ 1.03)	0.043
Model 3	1 (ref)	0.88 (0.64 ∼ 1.22)	0.87 (0.64 ∼ 1.18)	0.76 (0.56 ∼ 1.04)	0.98 (0.94 ∼ 1.03)	0.101

Values are n or hazard ratio (95% confidence interval). Ref, reference. Model 1: crude model. Model 2: adjusted for age, gender, and race or ethnicity. Model 2: adjusted for gender, age, race/ethnicity, PIR, educational status, marital status, BMI, HbA1c, serum total cholesterol, diabetes medication use, diabetes duration, Insulin therapy, Serum cotinine, calcium, serum triglycerides, urine albumin, magnesium, vitamin D, alcohol intake, multivitamin supplements use, kidney disease, TEI, HEI, HOMA-IR, hypertension, hyperlipidemia, CVD, smoke, physical activity

In the sensitivity analyses, these associations were largely unchanged when patients who died early were excluded, dietary protein intake was considered, patients with liver disease were excluded, and the association among patients with normal BUN was analyzed. After IPTW, the HRs of all-cause and CVD mortality remained largely unchanged when compared with that after the primary analyses (Supplemental Tables S1 and S2).

**Fig. 1:**
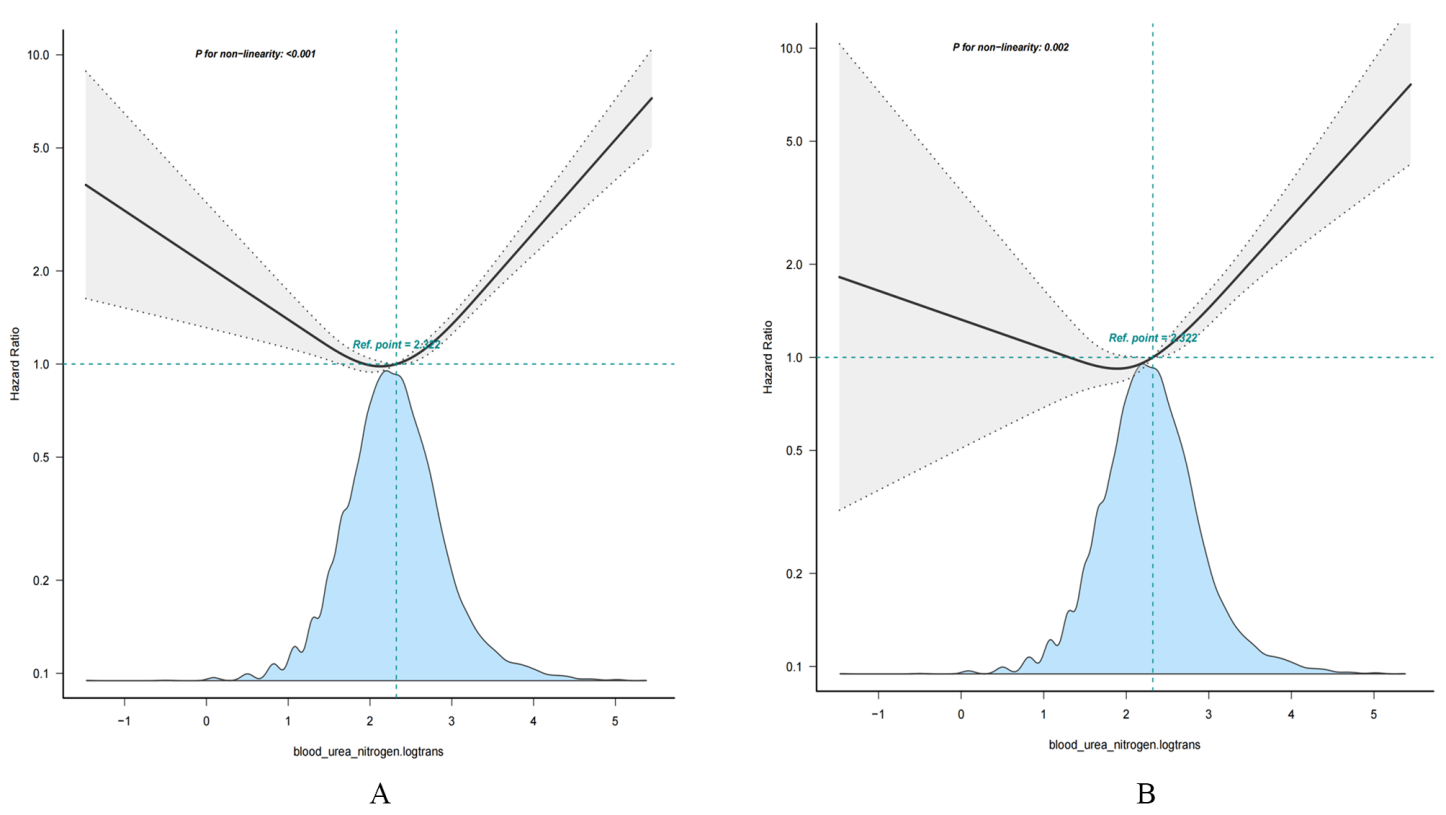
Smooth curve fitting demonstrates the relationship between BUN levels after log2-transformation and risk of all-cause and CVD mortality. **A**: all-cause mortality; **B**: CVD mortality. The predicted risk for all-cause and CVD mortality in the y-axis and the BUN levels after log2 transformation in the x-axis. The black line and gray area represent the estimated values and their corresponding 95% confidence intervals, respectively. The adjused covariates included gender, age, race/ethnicity, PIR, educational status, marital status, BMI, HbA1c, serum total cholesterol, diabetes medication use, diabetes duration, Insulin therapy, Serum cotinine, calcium, serum triglycerides, urine albumin, magnesium, vitamin D, alcohol intake, multivitamin supplements use, kidney disease, TEI, HEI, HOMA-IR, hypertension, hyperlipidemia, CVD, smoke, physical activity

A nonlinear association was found between log2-transformed BUN and all-cause and CVD mortalities in the results of the generalized additive models and smooth curve fittings. Both curves of all-cause and CVD mortalities showed a downward slope in HRs when the BUN level was < 2.322 mmol/L and an ascending slope in HRs when the BUN level was > 2.322 mmol/L (Fig. 1). The results of the threshold effect analysis indicated that BUN was negatively correlated with all-cause mortality when the BUN levels were less than the turning point; meanwhile, a positive correlation existed when the BUN levels were more than the turning point. However, BUN levels were not associated with CVD mortality when the levels were less than the turning point, while a positive correlation still existed when the BUN levels were more than the turning point. The inflection points were close to the BUN level of 5 mmol/L (Table 3).

**Table 3:** Threshold effect analysis of BUN levels on all-cause and CVD mortality based on segmented linear regression model

Outcome:	HR (95%CI)	*P*-value
**linear effect**	1.863 (1.804,1.922)	< 0.001
Inflection point	5 mmol/L	
< 5 mmol/L	0.602 (0.442,0.821)	0.0013
> 5 mmol/L	1.609 (1.46,1.774)	< 0.001
Log likelihood ratio test		< 0.001
**A**		
**Outcome**:	**HR (95%CI)**	***P*** **-value**
**linear effect**	1.366 (1.308,1.424)	< 0.001
Inflection point	5 mmol/L	
< 5 mmol/L	2.191 (0.424 ∼ 11.315)	0.349
> 5 mmol/L	1.428 (1.227 ∼ 1.662)	< 0.001
Log likelihood ratio test		< 0.34
**B**		

A: all-cause mortality; B: CVD mortality. Log likelihood ratio test results comparing linear regression model with two piece wise linear regression model adjusted for gender, age, race/ethnicity, PIR, educational status, marital status, BMI, HbA1c, serum total cholesterol, diabetes medication use, diabetes duration, Insulin therapy, Serum cotinine, calcium, serum triglycerides, urine albumin, magnesium, vitamin D, alcohol intake, multivitamin supplements use, kidney disease, TEI, HEI, HOMA-IR, hypertension, hyperlipidemia, CVD, smoke, physical activity

## Discussion

To the best of our knowledge, this study is the first to explore the correlation of BUN levels with CVD and all-cause mortality in patients with diabetes. In this large and nationally representative sample of US individuals with diabetes, a nonlinear association was observed between BUN and all-cause and CVD mortality. That is, the BUN levels should be controlled at appropriate levels, and significantly high and low BUN levels should be avoided to reduce the all-cause mortality. Moreover, higher BUN levels were positively associated with a higher prevalence of CVD mortality; hence, the BUN levels should be appropriately controlled.

Previous studies have also shown that BUN levels were correlated with the prevalence of all-cause and CVD mortalities. A previous cohort study of 252 participants with pulmonary disease conducted by Tatlisu et al. [24] concluded that high BUN levels were correlated with augmented risks of all-cause mortality in hospitalized and cardiogenic shock patients. Results from another prospective cohort study of 603 patients with CVD showed an association between BUN and long-term mortality [25]. However, the nonlinear association of BUN with mortality has not been explored in the aforementioned studies. Previous prospective cohort studies have shown a U-shaped association between BUN concentration or BUN/creatinine (Cre) ratio and mortality. One cohort study of 42,038 participants found a U-shaped relationship between BUN/Cre ratio and all-cause mortality in the general population, but the association of BUN/Cre ratio with CVD mortality had not been found after adjusting for potential covariates [26]. Some studies have reported that BUN can be used to predict heart disease [27–29]. Moreover, BUN is a more valuable predictor of acute coronary syndrome than Cre [30, 31]. Another cohort study of 26,835 Chinese individuals also showed a nonlinear association of BUN with CVD mortality, and both high and low levels of BUN increased the prevalence of stroke mortality [32]. Although BUN is a relatively well-known indicator, little is known about its optimal level for patients with diabetes. Our findings suggest that maintaining the BUN levels close to the inflection point may be beneficial for preventing CVD or all-cause mortality in patients with diabetes. Moreover, we found that low levels of BUN were not correlated with CVD mortality among US patients with diabetes, which could be explained by the discrepancy in the study population [33–35].

The correlation of BUN with the risk of mortality is biologically plausible. In particular, the disturbance in glucose homeostasis is the potential basis of the association of BUN with mortality. Abnormal BUN levels indicate renal function impairment, and the kidneys play an important role in maintaining glucose homeostasis [36, 37]. Uremic metabolites (including urea and sulfates) can disrupt glucose homeostasis [9]. Experimental evidence also suggests that urea may be responsible for abnormal insulin levels [7, 8]. In addition to reflecting kidney function, elevated BUN levels may indicate a decrease in the amount of water in the body, thereby increasing the risk of stroke [38]. Furthermore, BUN levels are associated with neurohormone activation [39]. At the onset of acute coronary syndrome, the renal angiotensin system and sympathetic nervous system are activated, thus increasing the reabsorption of BUN [40].

This study has several major strengths. Most importantly, we used data from a large representative sample of the US population with a relatively long follow-up time; this study is the first to explore the relationship of BUN with mortality in patients with diabetes. Moreover, considering the profile of BUN levels, we additionally adjusted for dietary protein, excluded individuals with liver disease, and performed the analysis among patients with normal BUN values. In addition, using the well-documented variables in the NHANES database, we adjusted for several diabetes-related factors, including HOMA-IR, HbA1c levels, diabetes duration, medication use, and serum cotinine level.

Despite these strengths, the following limitations were considered. First, the causality of BUN on mortality is unclear based on the present study; hence, future studies should examine potential underlying mechanisms this causality. Second, as the NHANES only collected the baseline values of BUN from 1999 to 2019, changes in BUN levels during follow-up could not be obtained, and repeated measurements or the average values of BUN should be considered in later studies. Third, we excluded the influence of some factors on BUN levels, such as dietary protein and liver disease; however, residual factors exist. However, as part of the sensitivity, BUN within the normal range was still associated with mortality. Fourth, although we included several covariates, we could not adjust for all residual covariates. However, stratified analyses were performed to make the findings robust and to confirm their heterogeneity. The IPTW method was also introduced to address the potential covariates. Fifth, the types of diabetes were not well distinguished in NHANES. However, the findings from this study were likely more representative of individuals with type 2 diabetes, because it included participants aged 20 years or older. Sixth, our findings are based on US citizens with diabetes, and the limitation to generalizability should also be considered.

## Conclusions

The nonlinear association of BUN with all-cause and CVD mortality exists in this large cohort of US patients with diabetes. Maintaining appropriate BUN levels may be beneficial to prevent all-cause or CVD mortality among patients with diabetes. Further clinical trials are also warranted to validate these results.

### Electronic supplementary material

Below is the link to the electronic supplementary material.

Supplementary Material 1

Supplementary Material 2

Supplementary Material 3

Supplementary Material 4

Supplementary Material 5

Supplementary Material 6

## Data Availability

The data underlying this article will be shared on reasonable request to the corresponding author.

## Abbreviations

CVD: Cardiovascular disease
Hb1Ac: Glycohaemoglobin
NCHS: National center for health statistics
NHANES: National health and nutrition examination survey
BMI: Body mass index
