# Supplementary material for: The long-term effects of blood urea nitrogen levels on cardiovascular disease and all-cause mortality in diabetes: a prospective cohort study
Source: BMC Cardiovasc Disord. 2024 May 16;24:256. doi: 10.1186/s12872-024-03928-6 (PMC11097526; doi:10.1186/s12872-024-03928-6)
Supplement: Supplementary file 1 — Supplementary Material 1 [file 12872_2024_3928_MOESM1_ESM.doc]

**Supplement Table 1. Sensitivity Analyses of all-cause mortality**

| **Analysis** | **N (%)^c^** | **HR** | **95% CI** | ***P*-value** |
| --- | --- | --- | --- | --- |
| **Excluding participants who died within 2 years of follow-up^a^** |  |  |  |  |
| BUN levels | 2089 (21.7) | 1.07 | 1.06–1.09 | <0.001 |
| **Participants within the normal range of BUN (3.1 to 9.5 mmol/L)^a^** |  |  |  |  |
| BUN levels | 1917 (21.7) | 1.05 | 1.02–1.08 | 0.002 |
| **considering dietary protein intake^a^** |  |  |  |  |
| BUN levels | 2441 (23.2) | 1.06 | 1.04–1.08 | <0.001 |
| **excluded participants with** **liver disease^a^** |  |  |  |  |
| BUN levels | 2290 (23.2) | 1.06 | 1.04–1.08 | <0.001 |
| **Inverse probability treatment weighted analyses^b^** |  |  |  |  |
| Q1(**<3.93**) of BUN levels | 339 (14.2) | Reference |  |  |
| Q4(**>6.43**) of BUN levels | 1105 (39.3) | 1.31 | 1.18–1.46 | <0.001 |

Abbreviations: HR, Hazard ratio; CI, confidence intervals.

^a^Adjusted for gender, age, race/ethnicity, PIR, educational status, marital status, BMI, HbA1c, serum total cholesterol, diabetes medication use, diabetes duration, Insulin therapy, Serum cotinine, calcium, serum triglycerides, urine albumin, magnesium, vitamin D, alcohol intake, multivitamin supplements use, kidney disease, TEI, HEI, HOMA-IR, hypertension, hyperlipidemia, CVD, smoke, physical activity.

^b^The variables included in the model were gender, age, BMI, race/ethnicity, PIR, alcohol intake, HOM_IR, smoking status, HbA1c, physical activity, diabetes duration, and kidney disease.

^c^N (%): “N” is the total number of all-cause mortality. “%” means the ratio of all-cause mortality.
