# Supplementary material for: The long-term effects of blood urea nitrogen levels on cardiovascular disease and all-cause mortality in diabetes: a prospective cohort study
Source: BMC Cardiovasc Disord. 2024 May 16;24:256. doi: 10.1186/s12872-024-03928-6 (PMC11097526; doi:10.1186/s12872-024-03928-6)
Supplement: Supplementary file 3 — Supplementary Material 3 [file 12872_2024_3928_MOESM3_ESM.doc]

**A**


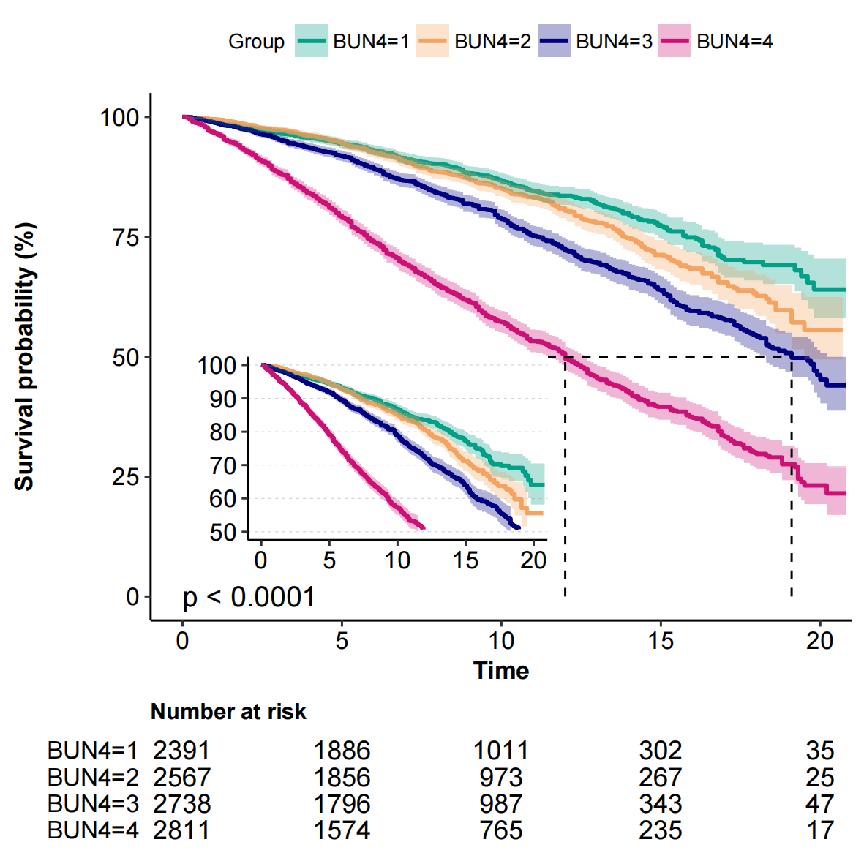


**B**

**
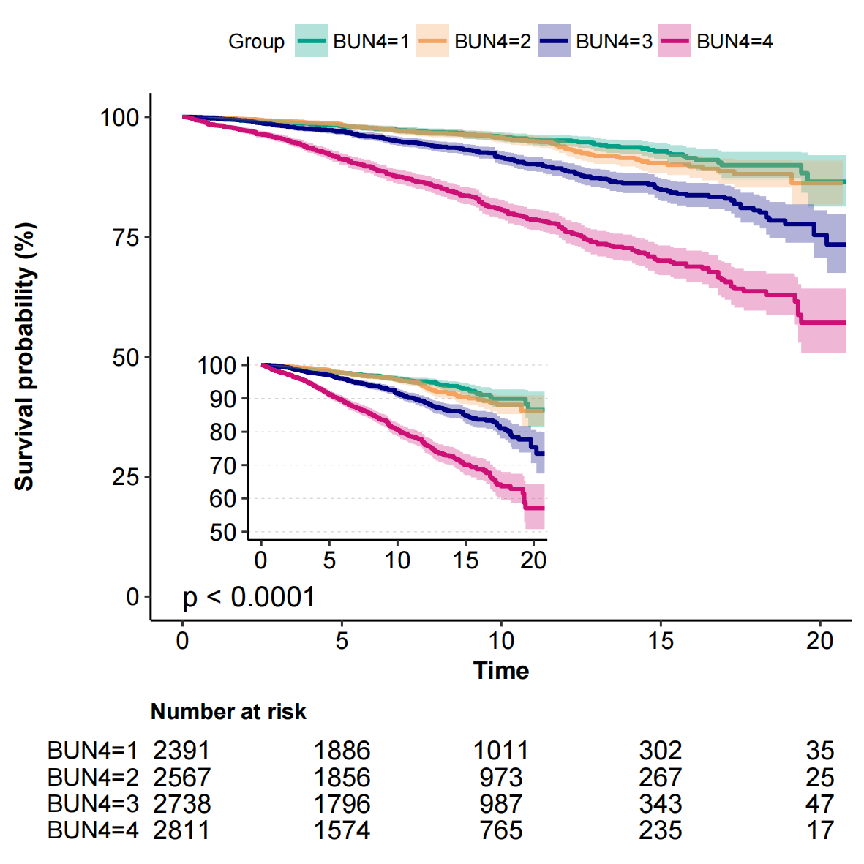
**

**C**


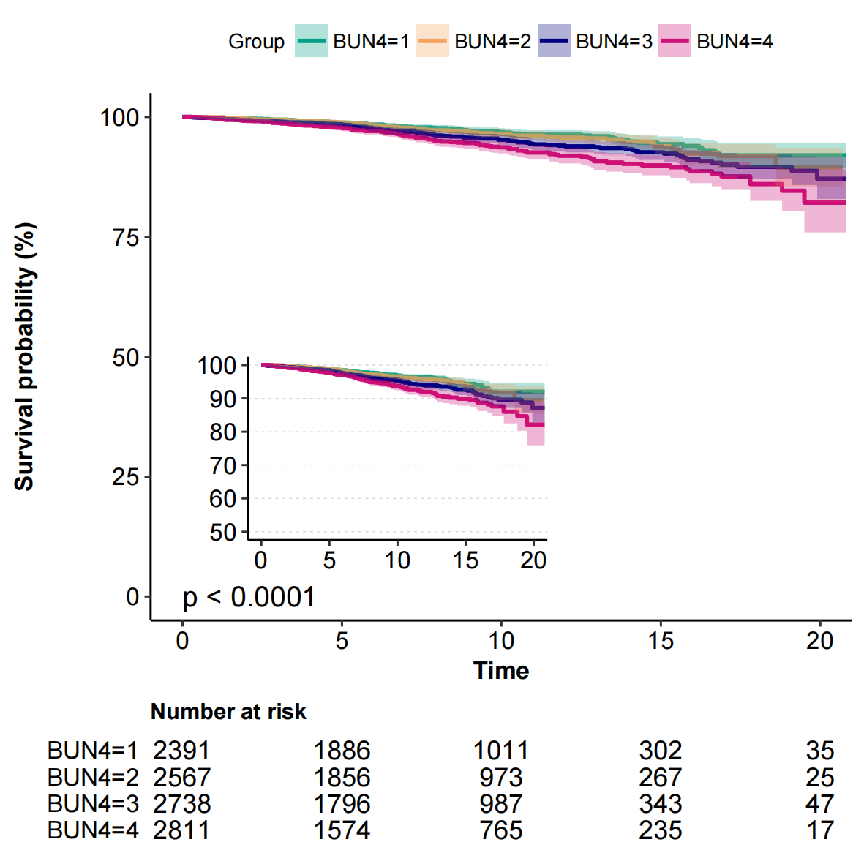


**Supplemental Fig. S2. The Kaplan–Meier analysis between the BUN levels quartiles and all-cause mortality (A), cardiovascular mortality (B) and cancer mortality (C).** BUN4 represented BUN quartiles. The P values were calculated by the log-rank test.
