# Supplementary figures and images for: The long-term effects of blood urea nitrogen levels on cardiovascular disease and all-cause mortality in diabetes: a prospective cohort study
Source: BMC Cardiovasc Disord. 2024 May 16;24:256. doi: 10.1186/s12872-024-03928-6 (PMC11097526; doi:10.1186/s12872-024-03928-6)

**Supplemental Figs. S3 Subgroup analysis of all-cause mortality.**


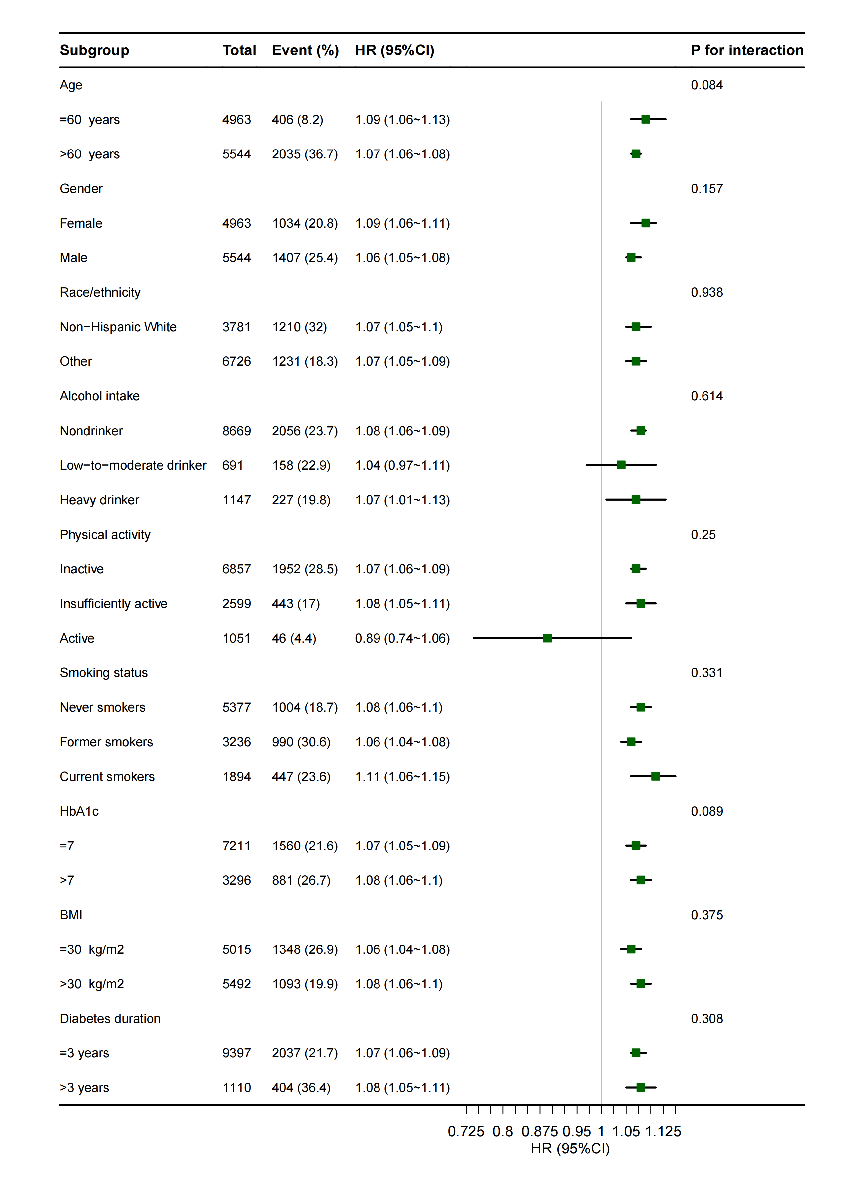

Supplement: Supplementary file 4 — Supplementary Material 4 [file 12872_2024_3928_MOESM4_ESM.doc]

**Supplemental Figs. S4 Subgroup analysis of CVD mortality.**


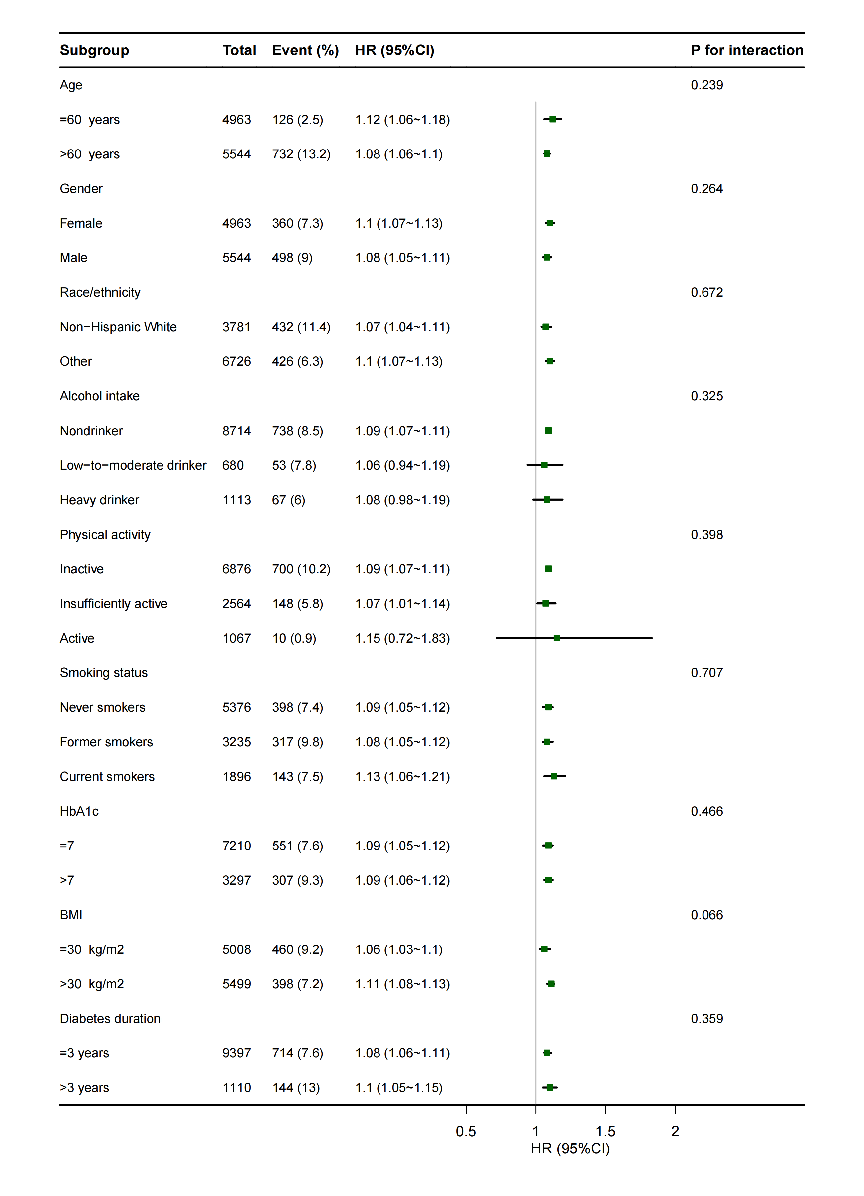

Supplement: Supplementary file 5 — Supplementary Material 5 [file 12872_2024_3928_MOESM5_ESM.doc]

**Supplemental Fig. S1 Flow chart of participants in this study.**


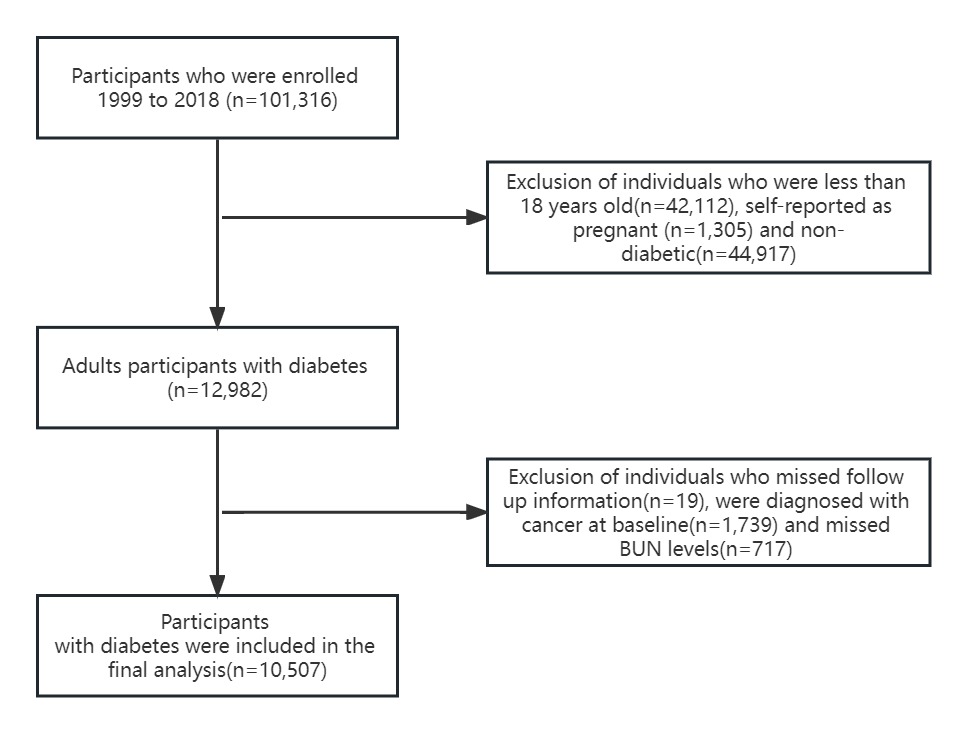

Supplement: Supplementary file 6 — Supplementary Material 6 [file 12872_2024_3928_MOESM6_ESM.doc]
